# Surface wear of attachments in patients during clear aligner therapy: a prospective clinical study

**DOI:** 10.1186/s40510-023-00506-y

**Published:** 2024-02-19

**Authors:** Qiuying Li, Kai Yang

**Affiliations:** Department of Orthodontics, School of Stomatology, Capital Medical University, No.4, Tiantanxili, Beijing, China

**Keywords:** Wear, Attachment, Clear aligner, Intraoral scanning, Superimposition

## Abstract

**Background:**

This prospective clinical study aimed to quantitatively evaluate the surface wear of attachments and investigate the associated risk factors. Additionally, the wear values and regions of three types of commonly used attachments were explored.

**Methods:**

Participants were recruited from the population of patients who received clear aligner therapy from October to December 2022. Intraoral scanning was performed on eligible participants before treatment (T0), immediately after initial bonding of attachments (T1), and at 2 months (T2), 4 months (T3), 6 months (T4), and 8 months (T5) after starting treatment. The attachment volume, average depth and regions of attachment wear were measured using superimposed digitized models. The Kruskal–Wallis test was performed to compare data between multiple groups. Multiple linear regression analyses were performed to evaluate risk factors for the volume of attachment wear.

**Results:**

A total of 47 patients with 617 attachments were included. As treatment time increased, the attachment volume decreased significantly (*P* = 0.003). The initial attachment volume was positively related to the volume of attachment wear (*β* = 0.527, *P* < 0.001). The volume of attachment wear was significantly greater in females than in males (*β* = 0.147, *P* = 0.020) and in optimized attachments than in conventional attachments (*β* = 0.308, *P* < 0.001). The wear of 3-mm rectangular attachments progressed from edges to buccal surfaces, with the deepest wear at corners of gingival edges; the wear of the optimized attachments was primarily located on surface ridges. The wear volume ratio of the optimized root control attachments was significantly greater than that of the 3-mm rectangular attachments at T3 (*P* = 0.011), T4 (*P* < 0.001), and T5 (*P* < 0.001).

**Conclusions:**

The volume of attachment wear increased gradually with treatment time. Sex, attachment type, and initial attachment volume were risk factors for the volume of attachment wear. The deepest wear regions of 3-mm rectangular attachments were at the corners of gingival edges, while the deepest wear regions of optimized attachments were at surface ridges. Four months after treatment, optimized root control attachments showed more relative wear than 3-mm rectangular attachments.

**Supplementary Information:**

The online version contains supplementary material available at 10.1186/s40510-023-00506-y.

## Background

Clear aligners have become prevalent in the treatment of different forms of malocclusion due to the development of thermoplastic materials and advancements in computer-aided design and manufacturing (CAD-CAM) technology [1]. Compared with traditional fixed appliances, clear aligners are considered more aesthetic, more comfortable, and more convenient for maintaining periodontal health [2, 3]. However, clear aligners still have some limitations in their efficacy when treatment involves complex tooth movements, such as rotation, torque, and extrusion, due to their mechanical properties. [4, 5].

To enhance the effectiveness of clear aligners, several auxiliary devices have been developed, and among them, attachments are one of the most potent. Attachments are composite resin buttons bonded to tooth surfaces that can assist with retention and improve the efficiency of tooth movements [6–8]. Materials for attachments should have sufficient aesthetic and mechanical properties. It is necessary for the material to both match the color of natural teeth and resist staining [9]. Furthermore, due to the frequent removal of aligners, the material needs to have good resistance to wear. Wear refers to the gradual loss and distortion of materials on solid surfaces. This phenomenon occurs due to the mechanical and/or chemical interactions between two surfaces in relative motion [10]. To ensure full utilization of attachments during orthodontic treatment, it is essential that their geometry and integrity are maintained consistently. The wear of attachments may impact anchorage control, thus affecting treatment outcomes [11].

The majority of current studies on the surface wear of attachments have been either in vivo qualitative studies or in vitro studies. Barreda et al. [12] used scanning electron microscopy to observe the surface wear of attachments over six months. They reported that surface wear occurred after six months of treatment and that Filtek Z350 XT (3 M ESPE, USA) composite resin exhibited better wear resistance. Lin et al. [13] performed visual inspection and reported that the first-year damage rate for attachments was approximately 12%. Chen et al. [14] tested the wear resistance of three kinds of composite resin used for attachments by an in vitro test. They concluded that Filtek Z350 XT Flowable (3 M ESPE, USA) composite resin showed greater volume loss. To date, few in vivo studies have been conducted to quantitatively evaluate the surface wear of attachments. A recent study used a three-dimensional (3D) model superimposition method to evaluate the surface wear of attachments in patients treated with clear aligner therapy [15]. However, the study only compared differences in surface wear and bond failure of different types of attachments. The values, regions, and risk factors for the surface wear of attachments during orthodontic treatment remain unclear.

The primary objectives of the current study were to quantitatively evaluate the surface wear of attachments in patients during clear aligner therapy and to investigate the risk factors related to attachment wear. The secondary objectives were to explore the regions and values of surface wear for three types of commonly used attachments (3-mm rectangular/optimized rotating/optimized root control) to provide theoretical support for future research.

## Material and methods

### Study participants

The participants of this prospective study were recruited among patients receiving orthodontic therapy with clear aligners from October to December 2022 in the Department of Orthodontics, Beijing Stomatological Hospital, Capital Medical University. The study protocol was approved by the Research Ethics Committee of Beijing Stomatological Hospital, Capital Medical University (CMUSH-IRB-KJ-PJ-2022–25), and prospectively registered with ChiCTR (registration number: ChiCTR2200064347). All participants signed informed consent forms. The inclusion criteria were as follows: (1) treatment with Invisalign® (Align Technology, USA); (2) permanent dentition; and (3) diagnosis of mild to moderate crowding based on model analysis (crowding degree < 8 mm). The exclusion criteria were as follows: (1) poor oral hygiene; (2) periodontal disease; (3) a habit of eating hard foods; (4) bruxism; (5) dental dysplasia; and (6) resin/crown restorations.

### Clear aligner therapy

All attachments for the included participants were bonded using Filtek Z350 XT (3 M ESPE, USA) composite resin according to the manufacturer’s instructions. All participants were instructed to change aligners every 7–14 days and wear them for at least 20–22 h per day. Aligners were to be removed before eating and replaced after tooth brushing. All participants received professional oral hygiene instructions. A two-month follow-up cycle was assigned for each participant.

### Data collection

Prior to orthodontic treatment, the following demographic and clinical data were collected: age, sex, dental malocclusion, vertical skeletal pattern, and anterior overbite. Dental malocclusion was classified as class I, II, or III according to the angle classification. The vertical skeletal pattern was classified by the Frankfort horizontal plane-mandibular plane (FH-MP) angle obtained through cephalometric analysis. FH-MP angles < 25.5°, 25.5°–36.7°, and > 36.7° were categorized as low, average, and high angles, respectively. The anterior overbite was classified as an open bite, a normal overbite, or a deep overbite based on the vertical distance of the maxillary anterior teeth covering the mandibular anterior teeth. Intraoral scanning with an iTero Element scanner (Align Technology, USA) was performed at various times: before treatment (T0), immediately after the initial bonding of attachments (T1), and at 2 months (T2), 4 months (T3), 6 months (T4), and 8 months (T5) after starting orthodontic treatment. All digitized models were saved in stereolithography (STL) format. The clinical information of the attachments, including arch, tooth position, and type, was noted. Lost attachments that were recorded during participants’ regular appointments were excluded from the final measurements.

### Model superimposition and measurement

**Fig. 1:**
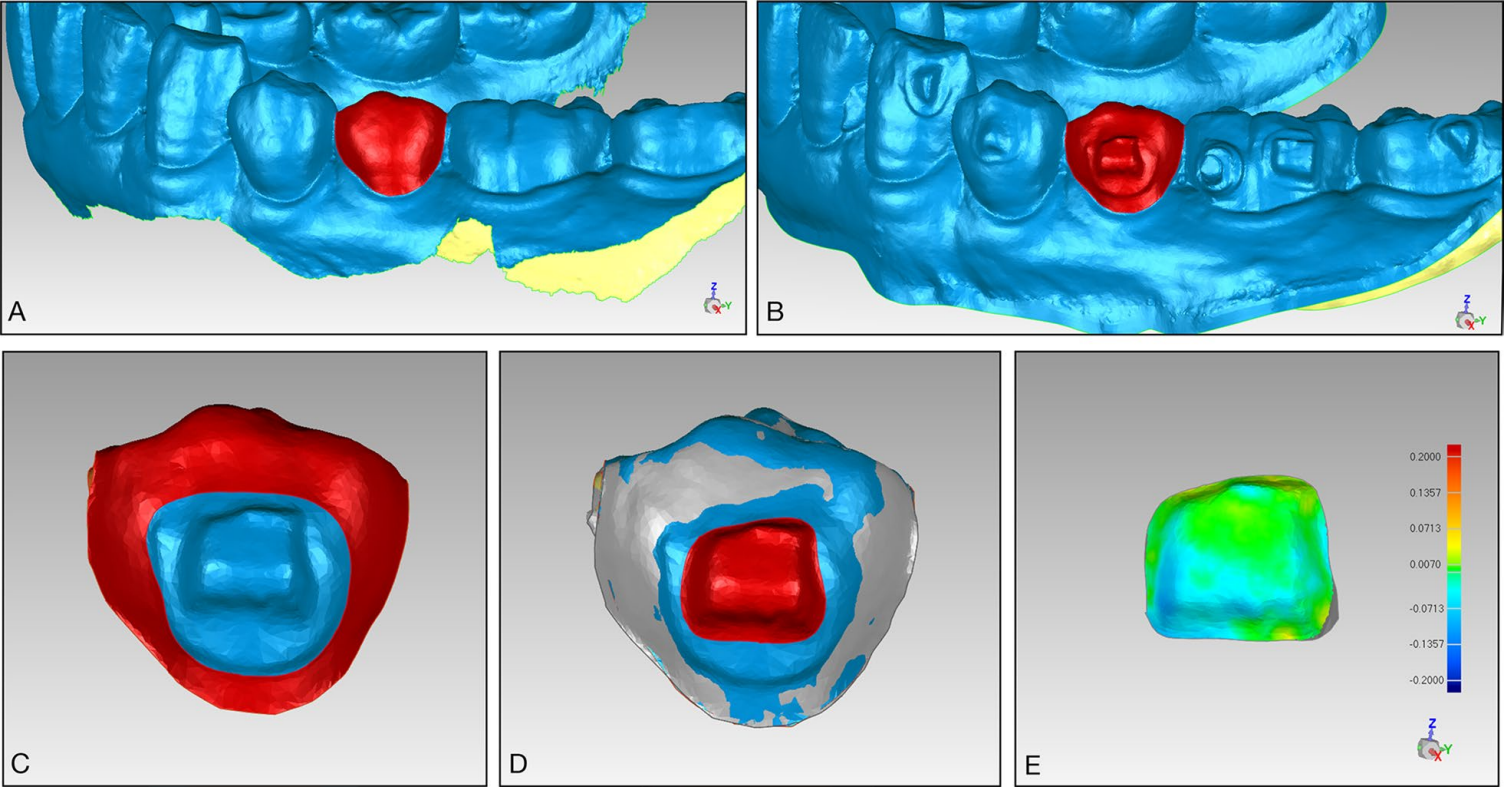
Superimposition and measurement of the models. **A** The target tooth without attachments was segmented from the whole dentition model (T0). **B** The target tooth with attachments was segmented from the whole dentition model (T1/T2/T3/T4/T5). **C** Tooth surfaces without resin were selected (T1/T2/T3/T4/T5). **D** A Boolean calculation was performed after the T0 (gray) and the T1/T2/T3/T4/T5 (blue) segmented models were superimposed based on the tooth surface without resin using the best-fit algorithm. The attachment area was selected to obtain the attachment volume at different follow-up times. **E** A 3D deviation analysis was performed after the T1 and T2/T3/T4/T5 segmented models were superimposed according to the above method. The segmented T1 model was set as a reference. The attachment area was selected to determine the average wear depth and wear area. The color map shows the morphological differences. The green color represents no differences. The yellow color represents the surface of the reference model (T1) on the inner side, which probably indicates the existence of plaque. The blue color represents the surface of the reference model (T1) on the outer side, which indicates that wear has occurred. The extent of wear is proportional to the darkness of the blue

All digitized models were imported into Geomagic Studio 2014 (Geomagic Co., USA) to accomplish the superimposition, and all teeth with single attachments bonded were analyzed. A target tooth is taken as an example to illustrate the process of superimposition and measurement. The target tooth was segmented from the whole dental arch model, which was obtained at T0 (Fig. 1A) and T1/T2/T3/T4/T5 (Fig. 1B). The tooth surfaces without resin were selected as a reference for superimposition (Fig. 1C). The segmented T0 and T1/T2/T3/T4/T5 models were automatically superimposed based on the “best-fit matching” function provided by the software until the minimum distance between the reference surfaces was reached. About 30,000 triangular surfaces were analyzed and calculated during each superimposition. The root-mean-square (RMS) values ranged from 10 to 30 μm during the superimposition process. After superimposition, the superimposed models were imported into Geomagic Qualify 2014 (Geomagic Co., USA). A Boolean calculation was performed to subtract the segmented T0 model from the segmented T1/T2/T3/T4/T5 model. The attachment area was selected to obtain the attachment volume at different follow-up times using the volume calculation function provided by the software (Fig. 1D). The segmented T1 model was superimposed on the segmented T2/T3/T4/T5 model based on the above methods. The superimposed models were also imported into Geomagic Qualify 2014 (Geomagic Co., USA). The segmented T1 model was set as a reference. A 3D deviation analysis was automatically performed by the software. The attachment area was selected to determine the average wear depth and wear regions. The deviations between the superimposed models are illustrated with a color map (Fig. 1E). The green color represents no differences. The yellow color represents the surface of the reference model (T1) on the inner side, which probably indicates the existence of plaque. The blue color represents the surface of the reference model (T1) on the outer side, which indicates that wear has occurred. The extent of wear is proportional to the darkness of the blue.

The volume of attachment wear at different follow-up times was defined as the attachment volume loss (mm^3^) between T1 and T2/T3/T4/T5. The ratio of wear volume at different follow-up times was defined as (attachment volume loss at different follow-up times/initial attachment volume) * 100%. The 3D deviation analyses were conducted only on three types of commonly used attachments (3-mm rectangular/optimized rotating/optimized root control) to observe wear regions and measure the average wear depth. All measurements were conducted three times by the same operator at a 2-week interval, and the average values were calculated.

### Sample size calculation

The sample size calculation was conducted based on a previous pilot study (unpublished data). With a sample size of 47 participants, a paired t test was calculated to have 90% power in detecting an effect size of 0.43 at a significance level of 0.05.

### Statistical analysis

All statistical analyses were performed using IBM SPSS 26.0 for Mac (SPSS, USA). The interclass correlation coefficient (ICC) was used to evaluate intraoperator agreement. The Shapiro‒Wilk test was conducted to test the normality of the data. The continuous data are presented as the mean ± standard error of the mean (SEM). The median and Q1 and Q3 (interquartile range) were also given if the data were not normally distributed. Statistical analyses to compare multiple independent groups were conducted using the Kruskal‒Wallis test. Multiple linear regression analyses (stepwise method) were conducted to determine the associated risk factors for the wear volume of attachments. The potential risk factors were age, sex, dental malocclusion, vertical skeletal pattern, anterior overbite, arch, tooth position, attachment type, and initial attachment volume. The criteria for inclusion and exclusion of the variables in the regression model were *α *< 0.05 and *α *> 0.1, respectively. A *P* value less than 0.05 was considered statistically significant.

## Results

Fifty-five patients were initially eligible for the study. Ultimately, 47 patients were included in the final analysis because 8 patients were unable to attend regular follow-up visits. Table 1 shows the clinical characteristics of the included participants. Throughout the 8-month follow-up period, 105 attachments were lost, and ultimately, 617 attachments were included in the final analysis. The characteristics of the included attachments are provided in Additional File 1.

**Table 1:** Clinical characteristics of included participants

Variable	Mean, n	SEM, %
*Continuous variables*		
Age (years)	26.77	8.45
*Categorical variables*		
Sex		
Male	14	29.79%
Female	33	70.21%
Dental malocclusion		
Class I	21	44.68%
Class II	15	31.91%
Class III	11	23.40%
Vertical skeletal pattern		
Low angle	8	17.02%
Average angle	19	40.43%
High angle	20	42.55%
Anterior overbite		
Open bite	3	6.38%
Normal overbite	12	25.53%
Deep overbite	31	65.96%

*SEM* Standard error of the mean

The ICC for attachment volume was 0.998, indicative of high intraoperator agreement. After 8 months of treatment, the median wear volume and wear volume ratio of the attachments was 0.813 mm^3^ and 13.72%, respectively. As the treatment time increased, the attachment volume decreased significantly (*P* = 0.003), while the wear volume and wear volume ratio of the attachments increased significantly (*P* < 0.001). The wear volume of the attachments at different follow-up stages is summarized in Table 2.

**Table 2:** Wear volume of attachments during the follow-up period

Time	Attachment volume (mm^3^)			Wear volume (mm^3^)			Wear volume (%)		
	Mean ± SEM	Median (Q1, Q3)	*P* value	Mean ± SEM	Median (Q1, Q3)	*P* value	Mean ± SEM	Median (Q1, Q3)	*P* value
T1	6.205 ± 0.144	6.565, (4.475,7.615)	0.003**	–	–	–	–	–	–
T2	5.920 ± 0.143	6.619, (4.233,7.301)		0.284 ± 0.019	0.194, (0.096,0.396)	< 0.001***	5.10 ± 0.38	3.34, (1.64,6.69)	< 0.001***
T3	5.722 ± 0.141	6.020, (4.045,7.063)		0.483 ± 0.022	0.411, (0.259,0.635)		8.52 ± 0.42	7.16, (4.37,11.26)	
T4	5.516 ± 0.138	5.750, (3.885,6.830)		0.689 ± 0.028	0.587, (0.409,0.853)		12.01 ± 0.52	12.01, (7.01,15.07)	
T5	5.280 ± 0.137	5.485, (3.668,6.626)		0.925 ± 0.032	0.813, (0.586,1.190)		16.20 ± 0.62	13.72, (10.24,19.77)	

*SEM* Standard error of the mean; *Q1, Q3* Interquartile range

**P < 0.01; ***P < 0.001

The multiple linear regression model showed statistically significant results (*F* = 15.318, *P* < 0.001). The adjusted *R*^*2*^ was 0.167. After stepwise regression, age, dental malocclusion, vertical skeletal pattern, anterior overbite, arch, and tooth position were excluded from the model. Table 3 shows the results of the multiple linear regression. The most significant influencing factor for the wear volume of attachments was the initial attachment volume. The initial attachment volume was positively related to the volume of attachment wear (*β* = 0.527, *P* < 0.001). The volume of attachment wear was significantly greater in females than in males (*β* = 0.147, *P* = 0.020) and in optimized attachments than in conventional ones (*β* = 0.308, *P* < 0.001).

**Table 3:** Results of multiple linear regression^†^ (stepwise method)

Independent variables	Regression coefficient (β)	SE	*t* value	*P* value
*Sex*				
Male	Reference			
Female	0.147	0.065	2.346	0.020*
*Attachment type*				
Conventional	Reference			
Optimized	0.308	0.074	3.871	< 0.001***
*Initial volume*	0.527	0.018	6.586	< 0.001***

^†^Dependent variable: wear volume of attachments

*P < 0.05; ***P < 0.001

**Fig. 2:**
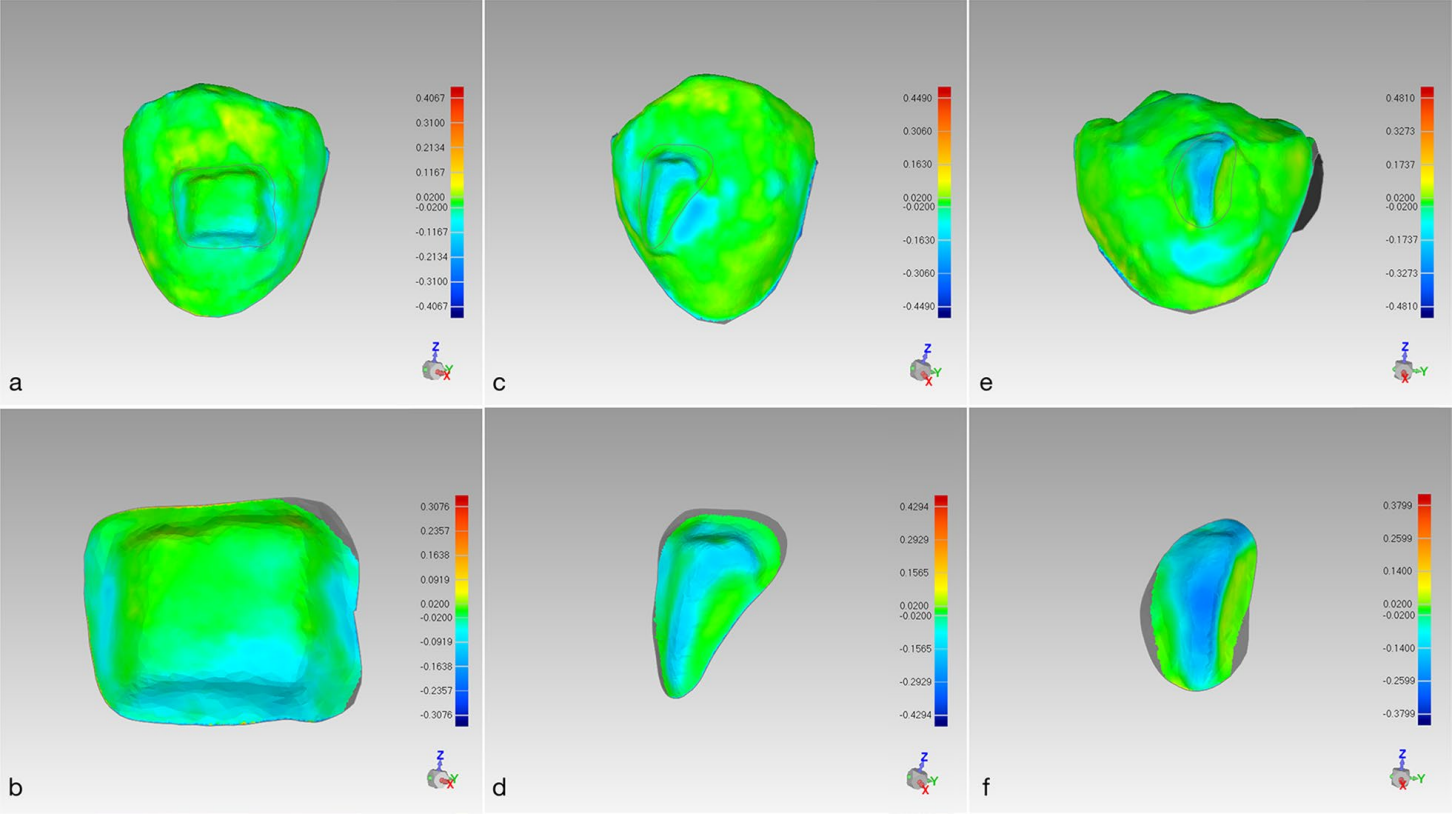
Color maps of three types of commonly used attachments generated from 3D deviation analyses. The 3-mm rectangular attachment (**a**) and its locally enlarged view (**b**). The optimized rotating attachment (**c**) and its locally enlarged view (**d**). The optimized root control attachment (**e**) and its locally enlarged view (**f**). The color map shows the morphological differences. The green color represents no differences. The yellow color represents the surface of the reference model (T1) on the inner side, which probably indicates the existence of plaque. The blue color represents the surface of the reference model (T1) on the outer side, which indicates that wear has occurred. The extent of wear is proportional to the darkness of the blue

**Fig. 3:**
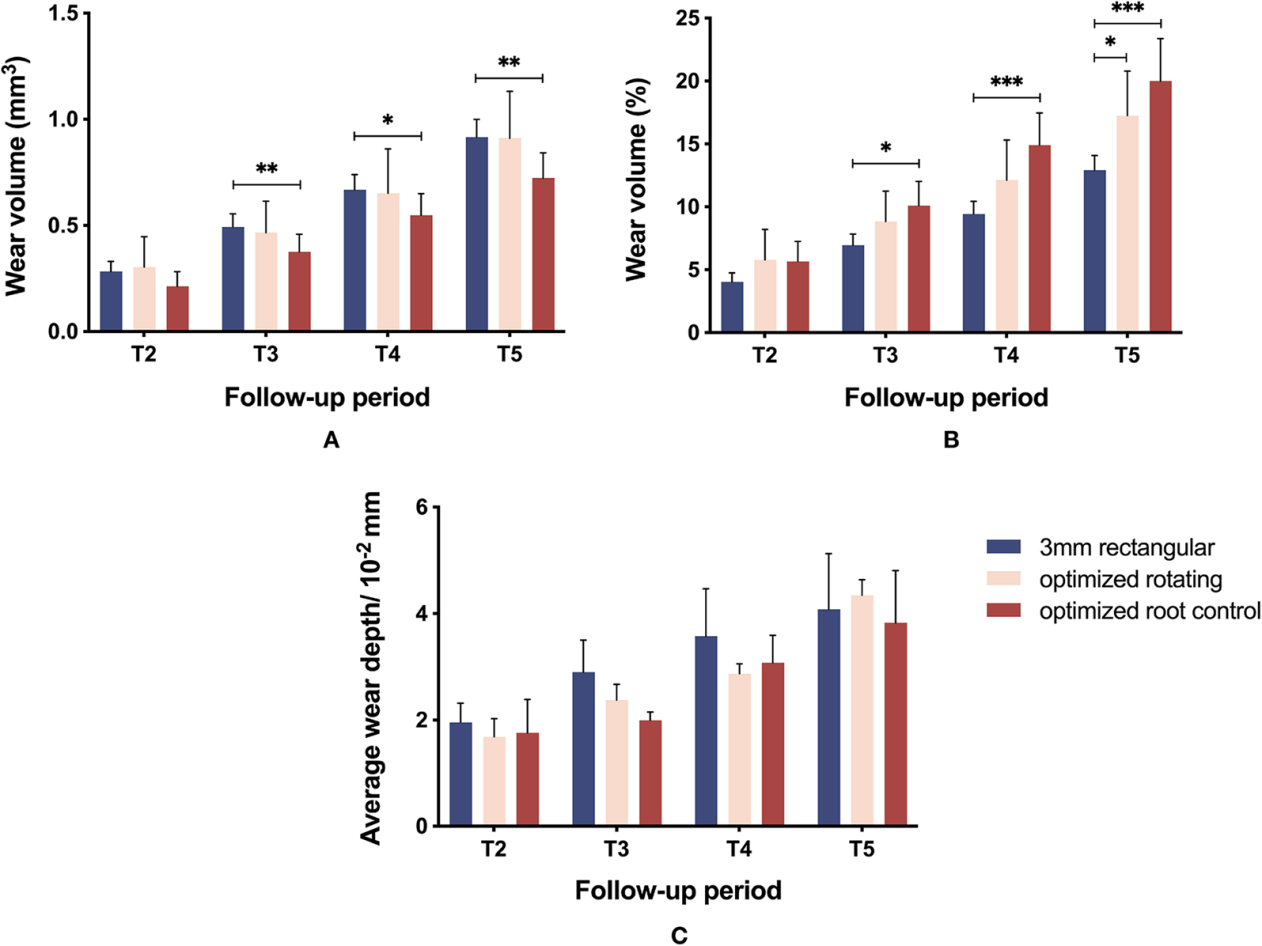
Comparison of wear between three types of commonly used attachments. The wear volume (**A**), the ratio of wear volume (**B**), and the average wear depth (**C**) are shown throughout the follow-up period. * indicates *P *< 0.05; ** indicates *P *< 0.01; *** indicates *P *< 0.001

Figure 2 shows the color maps of three types of commonly used attachments obtained by 3D deviation analyses after 8 months of treatment. The wear regions of 3-mm rectangular attachments progressed from edges to buccal surfaces, with the deepest wear at the corners of the gingival edges (Fig. 2a, b). The wear regions of the optimized rotating (Fig. 2c, d) and optimized root control (Fig. 2e, f) attachments were primarily located on the surface edge ridges. The wear volume of the 3-mm rectangular attachments was significantly greater than that of the optimized root control attachments at T3 (*P* = 0.009), T4 (*P* = 0.011), and T5 (*P* = 0.001) (Fig. 3A). The wear volume ratio of the optimized root control attachments was significantly greater than that of the 3-mm rectangular attachments at T3 (*P* = 0.011), T4 (*P* < 0.001), and T5 (*P* < 0.001), while the wear volume ratio of the optimized rotating attachments was significantly greater than that of the 3-mm rectangular attachments at T5 (*P* = 0.047) (Fig. 3B). However, the average wear depth of the three types of attachments did not show significant differences (*P* > 0.05) (Fig. 3C). The wear values of the three types of attachments are provided in Additional file 2.

## Discussion

This study demonstrated that the volume of attachment wear increased gradually as the treatment time increased. The median volume of attachment wear was 0.813 mm^3^ after 8 months of treatment. According to a previous study, the wear volume of Filtek Z350 XT composite resin utilized for producing attachments was approximately 0.75 mm^3^ in an in vitro wear test, which is comparable to our results [14]. However, Palaniappan et al. [16] evaluated the wear of several composite resins used for posterior restorations. The 1-year surface volume loss of restorations was found to be approximately 0.15 mm^3^, lower than that reported in our study. There are several possible explanations for this result. First, the wear of attachments under clinical conditions may be impacted by multiple factors, such as tooth brushing, eating, occlusal force, aligner removal, and the oral environment. Patients treated with clear aligners have to brush their teeth more often than individuals with just posterior restorations, and they also need to remove their aligners frequently. More frequent friction may have resulted in greater wear of attachments. Second, the variation in the wear volume between studies could be attributed to the differences in composite resin characteristics and types. The wear resistance of composite resin is influenced by the size, quantity, geometry, and distribution of fillers, the type of monomers, and the bond between the organic matrix and fillers [10]. Previous studies have reported that the wear resistance of composite resin could be improved by reducing the size and the distance between filler particles and increasing the bonding strength between the matrix and the filler [17, 18]. Moreover, the differences between studies may also be associated with the precision of scanners, methods for superimposition and measurement, and the accuracy of the superimposition software [19]. Previous clinical trials investigating the wear of composite resin often made impressions and replicas first, which were then scanned with an optical scanner for superimposition and measurement [16, 19]. Replication procedures could cause errors that may influence the measurement of wear. With the emergence and application of optical impression technology, clinicians can obtain digital models more conveniently through intraoral scanning. Several studies in the field of orthodontics have utilized the iTero scanner to acquire digitized models for measurement [14, 20]. According to previous studies, the mean precision of the iTero Element scanner ranged from 20 to 50 μm, which is acceptable for research purposes [21, 22]. This study incorporated previous research methods using Geomagic software to superimpose the 3D models for volumetric measurement and 3D deviation analysis [14, 23, 24]. This method can not only obtain wear values but also allow the observation of wear regions. When using software for 3D superimposition, the “best-fit matching” function employs a high-level, freeform surface-based registration method called the iterative closest point (ICP) algorithm, proposed by Besl and McKay [25]. This method can clearly reveal the goodness-of-fit (RMS) values. A deviation of less than 10 μm indicates an excellent fit, while a deviation greater than 50 μm indicates a poor fit [26]. In this study, the standard deviation of the reference surfaces during superimposition ranged from 10 to 30 μm, which is acceptable.

Our previous study assessed risk factors for attachment loss, revealing that arch and tooth position significantly impacted attachment loss. Attachments in the mandibular arch and molars exhibited higher loss rates [27]. However, regarding risk factors for the wear volume of attachments, the final regression model included three variables: sex, attachment type, and initial attachment volume. In the final model, the initial attachment volume (*β* = 0.527, *P* < 0.001) contributed the largest amount of explained variance, followed by the attachment type (*β* = 0.308, *P* < 0.001) and sex (*β* = 0.147, *P* = 0.020). Taken together, these variables accounted for 16.7% of the explained variance in the wear volume of attachments. It is expected that attachments with a larger initial volume will exhibit a greater wear volume since there is a greater surface area for relative friction. Our results showed that the optimized attachments were prone to wear. A possible explanation for this might be that optimized attachments have active surfaces that can exert precise biomechanical forces on teeth [28, 29]. One intriguing finding of this study was that females were observed to have a greater volume of attachment wear than males. According to McGrath et al. [30], females are more likely to perceive oral health as improving their quality of life, mood, appearance, and general well-being. Several studies have reported that females are more prone to experiencing oral impacts than males [31, 32]. Based on these findings, we speculated that female patients may exhibit greater frequency, duration, and care of brushing during orthodontic treatment, along with a higher frequency of aligner removal, resulting in a greater volume of attachment wear.

According to color maps generated from 3D deviation analyses, the corners of the gingival edges were the region of deepest wear of 3-mm rectangular attachments. It is possible that the reason for this is that aligners were first dislocated from the gingival side, causing the gingival edges of attachments to be subjected to greater friction. The wear regions of the two optimized attachments were primarily located on the surface edge ridges, possibly as a result of the presence of active surfaces. This study revealed that after 4 months of orthodontic treatment, the 3-mm rectangular attachments exhibited a significantly greater wear volume and smaller wear volume ratio than the optimized root control attachments. The regression model results suggested that the wear volume was more strongly affected by the initial attachment volume than the attachment type. The 3-mm rectangular attachments had noticeably larger initial volumes than the optimized root control attachments, which provides a reasonable explanation for the results. The optimized root control attachments had a smaller wear volume, but their relative wear was greater than that of the 3-mm rectangular attachments due to their smaller initial volume. This indicates the significance of assessing the wear of optimized root control attachments during the follow-up monitoring of patients undergoing clear aligner therapy, as excessive wear may adversely affect the retention of aligners and the efficiency of tooth movement.

There are some limitations to the current study that cannot be ignored. Since only one type of composite resin (Filtek Z350 XT) was used, it is not possible to generalize the results to all resins. Previous studies have indicated that aligner materials deliver decreased forces over time and show signs of aging [33, 34]. In this study, participants were instructed to change aligners every 7–14 days. Variations in the duration of wearing each set of aligners may impact the accuracy of the outcomes. Moreover, multiple linear regression was utilized to analyze the potential risk factors for the volume of attachment wear in this study. However, the sample size remains insufficient, and a larger sample size will provide more accurate results, thus enabling the analysis of additional potential risk factors. To obtain more accurate results, the accuracy of the oral scanner and the precision of the superimposition method need to be improved in future studies. It may be possible to overcome attachment wear by developing resins with better wear resistance. However, additional research is necessary to fully investigate this potential solution. Although we examined the surface wear of attachments, the influence of attachment wear on tooth movement remains unclear. Therefore, future research on this topic is recommended.

## Conclusions

The conclusions of this study can be summarized as follows:

1. After 8 months of treatment, the median wear volume and wear volume ratio of the attachments was 0.813 mm^3^ and 13.72%, respectively. As the treatment time increased, the attachment volume decreased, while the wear volume and wear volume ratio of the attachments increased.
2. The wear volume of attachments was not affected by age, dental malocclusion, vertical skeletal pattern, anterior overbite, arch, or tooth position. Attachments with larger initial volumes exhibited larger wear volumes. Moreover, the volume of attachment wear was greater in females than males and in optimized attachments than in conventional attachments.
3. The wear regions of 3-mm rectangular attachments progressed from edges to buccal surfaces, with the deepest wear at the corners of the gingival edges, while those of optimized rotating and root control attachments were primarily located on the surface edge ridges.
4. Four months after orthodontic treatment, the relative wear of the optimized root control attachments was greater than that of the 3-mm rectangular attachments. The surface wear of optimized root control attachments needs to be carefully monitored by clinicians to prevent excessive attachment wear from affecting the retention of aligners and the efficiency of tooth movement.

### Supplementary Information

**Additional file 1**: Characteristics of included attachments.

**Additional file 2**: Wear values of three types of commonly used attachments (Mean ± SEM).

## Data Availability

The datasets used and/or analyzed during the current study are available from the corresponding author on reasonable request.

## List of abbreviations

3D: Three dimensional
CAD-CAM: Computer-aided design and computer-aided manufacturing
FH-MP: Frankfort horizontal plane-mandibular plane
STL: Stereolithography
RMS: Root-mean-square
ICC: Interclass correlation coefficient
SEM: Standard error of the mean
ICP: Iterative closest point

## References

[1] Weir T (2017). Clear aligners in orthodontic treatment. Aust Dent J.

[2] Gao M, Yan X, Zhao R, Shan Y, Chen Y, Jian F (2021). Comparison of pain perception, anxiety, and impacts on oral health-related quality of life between patients receiving clear aligners and fixed appliances during the initial stage of orthodontic treatment. Eur J Orthod.

[3] Jiang Q, Li J, Mei L, Du J, Levrini L, Abbate GM (2018). Periodontal health during orthodontic treatment with clear aligners and fixed appliances: a meta-analysis. J Am Dent Assoc.

[4] Simon M, Keilig L, Schwarze J, Jung BA, Bourauel C (2014). Treatment outcome and efficacy of an aligner technique–regarding incisor torque, premolar derotation and molar distalization. BMC Oral Health.

[5] Rossini G, Parrini S, Castroflorio T, Deregibus A, Debernardi CL (2015). Efficacy of clear aligners in controlling orthodontic tooth movement: a systematic review. Angle Orthod.

[6] Dasy H, Dasy A, Asatrian G, Rózsa N, Lee HF, Kwak JH (2015). Effects of variable attachment shapes and aligner material on aligner retention. Angle Orthod.

[7] Simon M, Keilig L, Schwarze J, Jung BA, Bourauel C (2014). Forces and moments generated by removable thermoplastic aligners: incisor torque, premolar derotation, and molar distalization. Am J Orthod Dentofacial Orthop.

[8] Papadimitriou A, Mousoulea S, Gkantidis N, Kloukos D (2018). Clinical effectiveness of Invisalign(R) orthodontic treatment: a systematic review. Prog Orthod.

[9] Feinberg KB, Souccar NM, Kau CH, Oster RA, Lawson NC (2016). Translucency, stain resistance, and hardness of composites used for Invisalign attachments. J Clin Orthod.

[10] Dionysopoulos D, Gerasimidou O (2021). Wear of contemporary dental composite resin restorations: a literature review. Restor Dent Endod.

[11] Mantovani E, Castroflorio E, Rossini G, Garino F, Cugliari G, Deregibus A (2019). Scanning electron microscopy analysis of aligner fitting on anchorage attachments. J Orofac Orthop.

[12] Barreda GJ, Dzierewianko EA, Muñoz KA, Piccoli GI (2017). Surface wear of resin composites used for Invisalign® attachments. Acta Odontol Latinoam.

[13] Lin S, Huang L, Li J, Wen J, Mei L, Xu H (2021). Assessment of preparation time and 1-year Invisalign aligner attachment survival using flowable and packable composites. Angle Orthod.

[14] Chen W, Qian L, Qian Y, Zhang Z, Wen X (2021). Comparative study of three composite materials in bonding attachments for clear aligners. Orthod Craniofac Res.

[15] da Veiga F, Jardim A, Curado de Freitas J, Estrela C (2023). Surface wear and adhesive failure of resin attachments used in clear aligner orthodontic treatment. J Orofac Orthop.

[16] Palaniappan S, Bharadwaj D, Mattar DL, Peumans M, Van Meerbeek B, Lambrechts P (2009). Three-year randomized clinical trial to evaluate the clinical performance and wear of a nanocomposite versus a hybrid composite. Dent Mater.

[17] Turssi CP, Ferracane JL, Vogel K (2005). Filler features and their effects on wear and degree of conversion of particulate dental resin composites. Biomaterials.

[18] Nihei T, Dabanoglu A, Teranaka T, Kurata S, Ohashi K, Kondo Y (2008). Three-body-wear resistance of the experimental composites containing filler treated with hydrophobic silane coupling agents. Dent Mater.

[19] Lawson NC, Radhakrishnan R, Givan DA, Ramp LC, Burgess JO (2015). Two-year randomized, controlled clinical trial of a flowable and conventional composite in Class I restorations. Oper Dent.

[20] Yan X, Zhang X, Ren L, Yang Y, Wang Q, Gao Y (2023). Effectiveness of clear aligners in achieving proclination and intrusion of incisors among Class II division 2 patients: a multivariate analysis. Prog Orthod.

[21] Zimmermann M, Ender A, Mehl A (2020). Local accuracy of actual intraoral scanning systems for single-tooth preparations in vitro. J Am Dent Assoc.

[22] Kwon M, Cho Y, Kim DW, Kim M, Kim YJ, Chang M (2021). Full-arch accuracy of five intraoral scanners: in vivo analysis of trueness and precision. Korean J Orthod.

[23] Weckmann J, Scharf S, Graf I, Schwarze J, Keilig L, Bourauel C (2020). Influence of attachment bonding protocol on precision of the attachment in aligner treatments. J Orofac Orthop.

[24] D'Antò V, Muraglie S, Castellano B, Candida E, Sfondrini MF, Scribante A (2019). Influence of dental composite viscosity in attachment reproduction: an experimental in vitro study. Materials.

[25] Besl PJ, McKay ND (1992). A method for registration of 3-D shapes. IEEE Trans Patt Anal Machine Intell.

[26] Peters MC, Delong R, Pintado MR, Pallesen U, Qvist V, Douglas WH (1999). Comparison of two measurement techniques for clinical wear. J Dent.

[27] Li Q, Yang K (2023). Loss of attachments in patients during orthodontic therapy with clear aligners: a prospective clinical study. Orthod Craniofac Res.

[28] Inoue S, Yamaguchi S, Uyama H, Yamashiro T, Imazato S (2020). Influence of constant strain on the elasticity of thermoplastic orthodontic materials. Dent Mater J.

[29] Karras T, Singh M, Karkazis E, Liu D, Nimeri G, Ahuja B (2021). Efficacy of Invisalign attachments: a retrospective study. Am J Orthod Dentofacial Orthop.

[30] Mc Grath C, Bedi R (2000). Gender variations in the social impact of oral health. J Ir Dent Assoc.

[31] Rusanen J, Lahti S, Tolvanen M, Pirttiniemi P (2010). Quality of life in patients with severe malocclusion before treatment. Eur J Orthod.

[32] Ashari A, Mohamed AM (2016). Relationship of the Dental Aesthetic Index to the oral health-related quality of life. Angle Orthod.

[33] Skaik A, Wei XL, Abusamak I, Iddi I (2019). Effects of time and clear aligner removal frequency on the force delivered by different polyethylene terephthalate glycol-modified materials determined with thin-film pressure sensors. Am J Orthod Dentofacial Orthop.

[34] Fang D, Li F, Zhang Y, Bai Y, Wu BM (2020). Changes in mechanical properties, surface morphology, structure, and composition of Invisalign material in the oral environment. Am J Orthod Dentofacial Orthop.

