# Supplementary material for: Surface wear of attachments in patients during clear aligner therapy: a prospective clinical study
Source: Prog Orthod. 2024 Feb 19;25:7. doi: 10.1186/s40510-023-00506-y (PMC10874919; doi:10.1186/s40510-023-00506-y)
Supplement: Supplementary file 1 — Additional file 1: Characteristics of included attachments. [file 40510_2023_506_MOESM1_ESM.docx]

Additional file 1. Characteristics of included attachments

| **Variable** | **n** | **%** |
| --- | --- | --- |
| Arch |  |  |
| Maxillary | 355 | 57.54% |
| mandibular | 262 | 42.46% |
| Tooth position |  |  |
| Incisor | 81 | 13.13% |
| Canine | 142 | 23.01% |
| Premolar | 255 | 41.33% |
| Molar | 139 | 22.53% |
| Attachment type |  |  |
| Conventional | 256 | 41.49% |
| Optimized | 361 | 58.51% |
