# Supplementary material for: Surface wear of attachments in patients during clear aligner therapy: a prospective clinical study
Source: Prog Orthod. 2024 Feb 19;25:7. doi: 10.1186/s40510-023-00506-y (PMC10874919; doi:10.1186/s40510-023-00506-y)
Supplement: Supplementary file 2 — Additional file 2: Wear values of three types of commonly used attachments (Mean ± SEM). [file 40510_2023_506_MOESM2_ESM.docx]

Additional File 2. Wear values of three types of commonly used attachments (Mean ± SEM)

| **Time** | **Average wear depth (10^-2^ mm)** | | | | **Wear volume (mm^3^)** | | | | **Wear volume (%)** | | | |
| --- | --- | --- | --- | --- | --- | --- | --- | --- | --- | --- | --- | --- |
|  | 3mm rectangular | Optimized rotating | Optimized root control | *P* value | 3mm rectangular | Optimized rotating | Optimized root control | *P* value | 3mm rectangular | Optimized rotating | Optimized root control | *P* value |
| T2 | 1.96 ± 0.36 | 1.69 ± 0.34 | 1.76 ± 0.63 | 0.914 | 0.283 ± 0.024 | 0.304 ± 0.070 | 0.214 ± 0.034 | 0.159 | 4.05 ± 0.36 | 5.79 ± 1.18 | 5.67 ± 0.79 | 0.323 |
| T3 | 2.90 ± 0.60 | 2.38 ± 0.30 | 2.00 ± 0.15 | 0.316 | 0.493 ± 0.031 | 0.466 ± 0.073 | 0.376 ± 0.041 | **0.031*** | 6.97 ± 0.44 | 8.84 ± 1.18 | 10.12 ± 0.95 | **0.037*** |
| T4 | 3.58 ± 0.89 | 2.87 ± 0.19 | 3.08 ± 0.52 | 0.708 | 0.669 ± 0.036 | 0.651 ± 0.102 | 0.548 ± 0.051 | **0.025*** | 9.46 ± 0.49 | 12.13 ± 1.55 | 14.93 ± 1.26 | **0.001**** |
| T5 | 4.08 ± 1,05 | 4.35 ± 0.29 | 3.83 ±0.98 | 0.910 | 0.916 ± 0.042 | 0.911 ± 0.107 | 0.723 ± 0.059 | **0.005**** | 12.93 ± 0.58 | 17.25 ± 1.73 | 20.01 ± 1.68 | **<0.001***** |

SEM: standard error of the mean.

*: P < 0.05; **: P < 0.01; ***: P < 0.01.
